# The Ergogenic Effect of Recombinant Human Erythropoietin on V̇O_2_max Depends on the Severity of Arterial Hypoxemia

**DOI:** 10.1371/journal.pone.0002996

**Published:** 2008-08-20

**Authors:** Paul Robach, Jose A. L. Calbet, Jonas J. Thomsen, Robert Boushel, Pascal Mollard, Peter Rasmussen, Carsten Lundby

**Affiliations:** 1 Ecole Nationale de Ski et d'Alpinisme, Chamonix, France; 2 The Copenhagen Muscle Research Centre, Rigshospitalet, Copenhagen, Denmark; 3 Department of Anaesthesia, Rigshospitalet, Copenhagen, Denmark; 4 Department of Physical Education, University of Las Palmas de Gran Canaria, Las Palmas, Spain; 5 Department of Sport Science, University of Århus, Århus, Denmark; 6 Department of Biomedical Sciences, University of Copenhagen, Copenhagen, Denmark; 7 Laboratoire “Réponses cellulaires et fonctionnelles à l'hypoxie”, EA 2363, A.R.P.E., Université Paris 13, Bobigny, France; University of Arizona, United States of America

## Abstract

Treatment with recombinant human erythropoietin (rhEpo) induces a rise in blood oxygen-carrying capacity (CaO_2_) that unequivocally enhances maximal oxygen uptake (V̇O_2_max) during exercise in normoxia, but not when exercise is carried out in severe acute hypoxia. This implies that there should be a threshold altitude at which V̇O_2_max is less dependent on CaO_2_. To ascertain which are the mechanisms explaining the interactions between hypoxia, CaO_2_ and V̇O_2_max we measured systemic and leg O_2_ transport and utilization during incremental exercise to exhaustion in normoxia and with different degrees of acute hypoxia in eight rhEpo-treated subjects. Following prolonged rhEpo treatment, the gain in systemic V̇O_2_max observed in normoxia (6–7%) persisted during mild hypoxia (8% at inspired O_2_ fraction (F_I_O_2_) of 0.173) and was even larger during moderate hypoxia (14–17% at F_I_O_2_ = 0.153–0.134). When hypoxia was further augmented to F_I_O_2_ = 0.115, there was no rhEpo-induced enhancement of systemic V̇O_2_max or peak leg V̇O_2_. The mechanism highlighted by our data is that besides its strong influence on CaO_2_, rhEpo was found to enhance leg V̇O_2_max in normoxia through a preferential redistribution of cardiac output toward the exercising legs, whereas this advantageous effect disappeared during severe hypoxia, leaving augmented CaO_2_ alone insufficient for improving peak leg O_2_ delivery and V̇O_2_. Finally, that V̇O_2_max was largely dependent on CaO_2_ during moderate hypoxia but became abruptly CaO_2_-independent by slightly increasing the severity of hypoxia could be an indirect evidence of the appearance of central fatigue.

## Introduction

In a normoxic environment, recombinant human erythropoietin (rhEpo) has long been known for its ergogenic properties. Evidence of this is supported by numerous reports showing that prolonged rhEpo treatment induces a consistent increase in maximal oxygen uptake (V̇O_2_max) [1]–[4]. Furthermore, it has been shown that endurance performance is dramatically augmented after rhEpo treatment [5]. The ergogenic effect of rhEpo is primarily mediated by hematological changes, since rhEpo stimulates - as endogenous renal Epo does - the proliferation of erythroid progenitor cells that ultimately augments red blood cell production, total hemoglobin mass and arterial oxygen content [6], [7], both factors being key determinants of oxygen transport capacity and aerobic performance [8].

On the other hand, the ergogenic effect of rhEpo in a hypoxic environment is much less documented. While exercise capacity is consistently increased after rhEpo in normoxia, this is not the case with exercise in hypoxia, since neither rhEpo injections [9] nor autologous blood transfusion [10] were found to improve V̇O_2_max at altitudes above 4000 m, in spite of enhanced arterial O_2_ content (CaO_2_). Likewise, with altitude acclimatization, CaO_2_ and systemic oxygen delivery are restored close to sea-level values, however V̇O_2_max measured at high altitude is only barely ameliorated [11], [12]. The main mechanism of this dissociation appears to be of cardiovascular origin, involving a reduction in peak muscle blood flow with altitude acclimatization [11], [13] caused by a reduction in peak cardiac output and a redistribution of blood flow toward non-exercising tissues in response to higher CaO_2_ [12]. Alternatively, it has been argued that in hypoxia V̇O_2_max is not as tightly dependent on CaO_2_ as in normoxic conditions, but is rather limited by the pressure gradient driving diffusion from the capillaries to the muscle mitochondria, due to the reduced arterial oxygen pressure (PaO_2_) [14], [15]. Nevertheless, since previous studies examining the influence of CaO_2_ on the cardiovascular response to exercise have been carried out after prolonged exposure to hypoxia, where accelerated erythropoiesis and hypoxia coexist, it has not been possible to separate putative effects linked to altitude acclimatization from those elicited by the increase in CaO_2_.

The fact that recombinant Epo boosts maximal oxygen transport at sea level but not at high altitude implies the existence of an “altitude threshold” beyond which the ergogenic effect of rhEpo on V̇O_2_max evanishes. Determining this threshold may give further insights into the minimal level of hypoxia required to initiate some mechanisms counteracting the advantageous hematological adaptations conferred by rhEpo.

The first objective of this study was to determine how does the rhEpo-induced rise in V̇O_2_max vary with the severity of hypoxia. From the results obtained in response to this first question, our second objective was to gain insights into the mechanisms involved in the interactions between hypoxia, CaO_2_ and V̇O_2_max.

## Methods

### Subjects

Eight Caucasian healthy male volunteers participated in the study. Their characteristics (mean±SD) were age: 27±7 yr, height: 180±4 cm, body mass: 83±7 kg, and maximal oxygen uptake: 4.1±0.2 l.min^−1^. The subjects were active in recreational sport activities such as jogging 1–2 times per week. During the entire study period, the subjects were asked not to deviate from their normal lifestyle, and this was well accepted by all. Written informed consent to participate in the study was obtained from all subjects. This study was carried out according to the Declaration of Helsinki and was approved by the Ethical Committee of Copenhagen and Frederiksberg Counties, Denmark. All the study took place at sea level in Copenhagen, Denmark.

### Treatment with Recombinant Human erythropoietin (rhEpo)

After baseline measurements, treatment with rhEpo (NeoRecormon, Roche, Mannheim, Germany) was started and lasted for fifteen weeks. On each occasion, 5000 IU was injected as follows. First two weeks: one injection every second day; third week: three injections on three consecutive days; week four to fifteen: one injection every week. All injections occurred between 08:00 and 10:00 a.m., and were preceded by 30 min of supine rest. Two weeks prior to rhEpo treatment all subjects received 100 mg iron.day^−1^ orally, and this was maintained throughout the entire study period. The present rhEpo treatment is an exploratory model, which has been developed by the Copenhagen Muscle Research Centre both for physiological studies and also for doping testing purposes. Our rhEpo treatment, which aimed to mimic what could be a procedure of rhEpo misuse, was shown to increase red blood cell volume substantially (∼10%) while maintaining the hematocrit at around 50% (corresponding to the upper limit for entering some competitions) throughout the study period [6]. Furthermore, our rhEpo treatment was associated with normal circulating Epo levels during the “maintenance” period [16].

### Experimental setup

**Figure 1:**
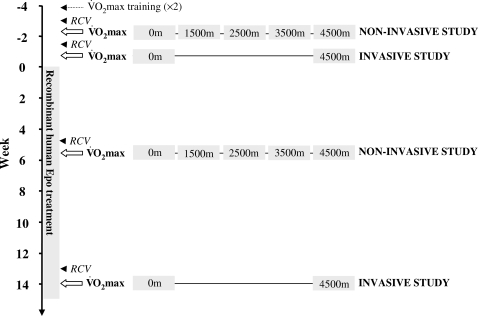
Experimental protocol. Summary of the experimental design, which involved two separate experiments in the same group of subjects: 1) a non-invasive study, and 2) an invasive study. The data on red blood cell volume (RCV) are available in a separate report [6].

The overall experiment design of the study is summarized on Fig. 1.

#### Non-invasive experiments

In order to gain insights into the altitude threshold beyond which the effect of rhEpo on maximal oxygen uptake becomes marginal, seven of the eight subjects (subjects #1 to #7) underwent a non-invasive experiment on two separate occasions, i.e. before and after five weeks of rhEpo treatment. On each occasion, the subjects performed an incremental cycling exercise until exhaustion in five conditions, i.e. in normoxia and in four levels of acute normobaric hypoxia equivalent to the altitudes of 1500 m (F_I_O_2_ = 0.174), 2500 m (F_I_O_2_ = 0.153), 3500 m (F_I_O_2_ = 0.134) and 4500 m (F_I_O_2_ = 0.115). In this study, the altitude of 1500 m refers to “mild hypoxia”, the altitudes of 2500–3500 m to “moderate hypoxia” and the altitude of 4500 m to “severe hypoxia”. The order between oxygenation conditions was randomly balanced in a single-blind manner. Each subject completed the five exercise tests over a period of 3–4 consecutive days with a maximum of two incremental tests per day, and if so with a recovery period of at least 120 min between the two tests. During baseline measurements, the non-invasive experiment (five tests) took place one week before the invasive experiment (two tests). On the experimental days the subjects reported to the laboratory at 08:00 a.m., and a catheter was inserted into an arm vein for lactate determination. Arterialized blood was obtained from a capillary blood sample drawn from a pre-warmed finger tip, by using an electrically heated pad (Heat Pad, OBH). Respiratory variables were measured continuously, as explained below.

Cardiac output was measured continuously during cycling exercise by a cardiac impedance technique (PhysioFlow PF-05, Manatec biomedical, Paris, France). The cardiac impedance device used in the present experiment has been previously validated during exercise in healthy subjects [17], [18]. This technique, which measures the changes in thoracic impedance during cardiac ejection in order to calculate stroke volume, has been described elsewhere [17]. Six thoracic impedance electrodes (Ag/AgCl, Hewlett Packard 40493 E) were placed after skin preparation as follow: four electrodes were placed at the base of the neck and along the xiphoid for impedance signal and two additional electrodes were placed for ECG signal. Resting arterial blood pressure, measured by automatic sphygmomanometry while the subject was sitting at rest on the ergometer, was used to calibrate the thoracic impedance device. Heart rate and stroke volume were continuously measured during the test. Cardiac output was averaged over 15-s intervals.

**Figure 2:**
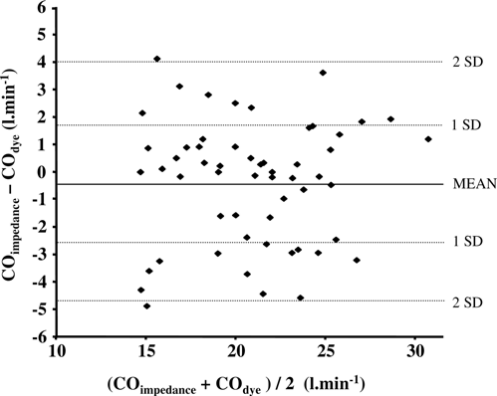
Agreement between impedance and dye dilution for cardiac output measurement. This graph shows the agreement (Bland-Altman plot) between the cardiac impedance technique and the indocyanine-green dye dilution method for measuring cardiac output during exercise obtained from 55 measurements in seven subjects. For each measurement, the difference between the two methods is plotted against the average of both techniques. The solid line indicates the mean bias, while the dotted lines indicate the 95% confidence intervals (2×standard deviation).

*Validity of cardiac impedance for cardiac output measurement*. In order to evaluate the validity of our cardiac impedance method for cardiac output measurement during the non-invasive study, cardiac output during incremental exercise in the invasive study was measured simultaneously by the cardiac impedance technique and the cardio-green dye dilution method. The agreement between the two techniques was assessed by the method of Bland and Altman [19]. The Bland-Altman plot (Fig. 2), obtained from 55 simultaneous measurements, shows that the mean bias was −0.41 l.min^−1^ with 95% confidence interval ranging between −4.83 and 4.01 l.min^−1^.

#### Invasive experiments

In order to gain insights into the control of maximal oxygen transport in hypoxia following prolonged rhEpo treatment, seven of the eight subjects (subjects #1–4 and #6–8) underwent an invasive experiment on two separate occasions, i.e. before and after fourteen weeks of rhEpo treatment. On each occasion, the subjects performed an incremental cycling exercise until exhaustion in two conditions, i.e. in normoxia and in normobaric hypoxia equivalent to an altitude of 4500 m (F_I_O_2_ = 0.115). As for the non-invasive experiment, here the altitude of 4500 m refers to “severe hypoxia”. The order between oxygenation conditions was randomly balanced in a single-blind manner and the recovery time between the two exercise bouts was 120 min. On the experimental days the subjects reported to the laboratory at 08:00 a.m., and catheters were placed under local anesthesia (2% Lidocain) [20]. Briefly, a 20 gauge catheter (Arrow, Ref. ES-14150, Reading, PA, USA) was inserted percutaneously using the Seldinger technique into the right femoral artery, 2–5 cm below the inguinal ligament and advanced 8 cm in the proximal direction. This catheter was connected to a blood pressure transducer positioned at the height of the fourth parasternal intercostal space (T100209A, Baxter, Unterschleissheim, Germany) and was also used to sample arterial blood. A similar catheter was inserted in the same femoral artery 1–2 cm below the previous catheter and advanced 5–10 cm in the proximal direction for cardiac output measurement. Another 20 gauge catheter (Arrow, Ref. ES-14150, Reading, PA, USA) was inserted in the right femoral vein, 2–3 cm below the inguinal ligament and advanced 8 cm in the distal direction, beyond the merger with the saphenous vein for femoral venous blood sampling. This catheter was also connected to a blood pressure transducer positioned at the height of the fourth intercostal parasternal space (T100209A, Baxter, Unterschleissheim, Germany) to measure femoral vein pressure. In the same femoral vein, a venous catheter with side holes (Radiopack TFE, Cook, Bjaerverskov, Denmark) was inserted and advanced ∼5 cm proximal to the inguinal ligament for the injection of ice-cold physiological saline solution. A thin polyethylene-coated thermistor (model 94-030-2.5F T.D. Probe, Edwards Edslab, Baxter, Irvine, CA, USA) was inserted through the venous catheter for blood flow measurements by the constant infusion thermodilution technique [21]. All these catheters were connected to three-way stopcocks and, along with the thermistor, sutured to the skin, under local anesthesia to minimize the risk of movement during exercise. An additional venous catheter was inserted into an antecubital vein to inject indocyanine green (ICG, Akorn Inc, IL) for measuring cardiac output by the dye-dilution method, as previously reported [22], [23]. A three-lead electrocardiogram (ECG) was displayed on a monitor during catheterization and the rest of the experimental procedures (Dialogue 2000, Danica, Copenhagen, Denmark). The ECG, blood pressure and the temperatures registered by the thermistor, as well as the infusate temperatures were recorded simultaneously with the data acquisition system (MacLab 16/s ADInstruments, Sydney, Australia). Cardiac output was measured continuously during exercise by the cardiac impedance technique. Respiratory variables were measured continuously, as explained below.

### Experimental protocol

#### Incremental exercise

The experimental protocol consisted of an incremental exercise bout during which the subjects cycled with a cadence close to 80 revolutions per minute on an electrically-braked ergometer (Excalibur, Lode, Gröningen, The Netherlands), first at 100 watts (W) during 15 min, then by increasing workload by 40 W every 90 seconds (s) until the subject could not sustain the pedaling pace. The maximal workload (W_max_) was calculated with the formula [24]: W_max_ = W_compl_+40×(t/90), where *W_compl_* is the last workload completed, *t* is the number of seconds that the final, not-completed workload was sustained and 40 (watts) is the workload increment. During the invasive experiment, a blood sample was obtained simultaneously from the femoral vein and artery, followed by the measurement of leg blood flow, and cardiac output at the 6^th^ and 12^th^ minutes at 100 W, within the last 45 s at each workload and at maximal exercise, as close as possible to exhaustion. In this specific study, only the measurements at the 12^th^ minute at 100 W and maximal exercise are reported. Also during normoxic trials the values at 260 W were given as an estimate of the corresponding peak workload attained in hypoxia (∼280 W). During the non-invasive experiment, a blood sample was obtained simultaneously from the forearm vein site and the capillary sampling site at the 12^th^ minute at 100 W and at maximal exercise. During all exercise tests, strong verbal encouragement was given to the subjects. The subjects practiced the incremental exercise until exhaustion twice before performing baseline experiments.

#### Normobaric hypoxia

Normobaric hypoxia was simulated by diluting ambient air with nitrogen via a mixing chamber, the dilution being continuously regulated via an oxygen-pressure probe (AltiTrainer 200, Sport and Medical Technology, Geneva, Switzerland). This device allows the inspired partial pressure of O_2_ (PO_2_) to be set a pre-determined level of normobaric hypoxia, the precision of the PO_2_ being ±0.82 mmHg. At rest and during exercise the subjects breathed through a face mask connected to two-way respiratory valve. The inspiratory port of the three-way valve was connected by a low resistance corrugated tube to a three-way stopcock hidden from the subjects, delivering either room air or normobaric hypoxia. During all the experiments in hypoxia, the subjects breathed hypoxic mixture at rest for 10 min before exercising.

#### Respiratory variables

Pulmonary V̇O_2_, CO_2_ production (V̇CO_2_), and expired minute ventilation (V̇_E_) were measured continuously using an automated metabolic cart (Quark b^2^, Cosmed Srl, Rome, Italy). Before each test ambient conditions were measured, then gas analyzer and flowmeter were calibrated by using high precision gases and a 3-litre syringe, respectively. The ventilatory variables were recorded as averages of 15 seconds. The highest 15-second measurement of V̇O_2_ was taken as representative of V̇O_2_max. The reason for using this short interval is that leg blood flow, leg V̇O_2_ and blood pressures were also assessed during a similar time interval.

#### Blood flow

Femoral venous blood flow was measured by constant-infusion thermodilution, as described in detail elsewhere [21]. Briefly, ice-cold saline was infused through the femoral vein simultaneously at flow rates sufficient to decrease blood temperature at the thermistor level by 0.5–1°C. Infusate and blood temperature were measured continuously during saline infusion (Harvard pump, Harvard Apparatus, Millis, MA, USA) via thermistors connected to the data acquisition system. Infusate temperature was measured with a thermistor set in a flow-through chamber (model 93-505, Edslab) connected to the venous catheter. Blood flow was calculated on thermal balance principles, as detailed by Andersen and Saltin [21].

#### Vascular Conductance

Systemic vascular conductance was calculated as the cardiac output divided by the mean arterial pressure. Leg vascular conductance was calculated as the quotient between leg blood flow and the pressure difference between the femoral artery and the femoral vein. All pressures were referred to the fourth parasternal intercostal space.

#### Blood samples and calculations

Blood was sampled anaerobically in heparinized syringes (or in capillary tubes (Radiometer) for the non-invasive experiment, according to the manufacturer's instructions) and immediately analyzed for hemoglobin concentration ([Hb]), hematocrit, oxygen saturation, blood pH, CO_2_ and O_2_ tensions, lactate, and potassium (ABL700 Series, Radiometer, Copenhagen, Denmark). Capillary hematocrit values obtained with the gas analyzer (ABL 700) were tested against the micro-centrifugation method during a separate procedure. A satisfactory agreement was found between the two methods (results not shown). Blood gases were corrected for measured femoral vein blood temperature (femoral venous and arterial blood gases). Plasma norepinephrine concentration was measured by ELISA (R&D Systems, Minneapolis, MN, USA). Blood O_2_ content (ml.l^−1^) in femoral artery and vein (CaO_2_ and C_fv_O_2_ respectively) was computed from the saturation and [Hb], i.e. (1.34×[Hb]×SO_2_)+(0.003×PO_2_). Systemic oxygen delivery was computed as the product of cardiac output and CaO_2_, while leg O_2_ delivery was obtained multiplying leg blood flow by CaO_2_. Leg V̇O_2_ was calculated using the direct Fick method, i.e. as the product of leg blood flow and arterial-venous difference in oxygen content. The systemic a-v O_2_ difference was calculated using the measured whole body V̇O_2_ and cardiac output (indirect Fick method). Leg lactate and potassium releases were calculated as the product of leg blood flow and the venous-arterial difference of lactate and potassium, respectively.

#### Red blood cell volume

Red blood cell volume was determined by a carbon monoxide rebreathing method twice before rhEpo treatment, and after 5 and 13 weeks of rhEpo treatment. This dataset has been reported elsewhere [6].

### Statistical analysis

The Kolmogorov-Smirnov's test was applied to examine the normality in the distribution of data. Due to the small number of participants in our study (n = 7), for each parameter, all the data that were obtained at a given power output (i.e. at 100 W or at peak exercise) were pooled before data distribution was examined (n = 28). The Bartlett's test was used to evaluate the uniformity of variance between conditions. If variance was not found to be homogeneous, ANOVA was replaced by the Welch's test. Once normality and variance homogeneity were verified, the differences in the measured variables among experimental conditions and exercise levels were analyzed using two-way ANOVA with repeated measures, with rhEpo and power output as within-subjects factors. Two separated ANOVA analyses were performed, one for testing the effect of rhEpo in normoxia and the other one for testing the effect of rhEpo in hypoxia. Another two-way ANOVA (rhEpo×hypoxia) was applied to the measured variables 1) at 100 W and 2) at peak exercise. When F was significant in the ANOVA, planned pair-wise specific comparisons were carried out using Student's paired *t* test adjusted for multiple comparisons with the Newman-Keul's procedure. Statistics were done with the Statview software, version 5.0. (SAS Institute, Cary, NC). The values are reported as arithmetic means±SE. Differences were considered as significant for P<0.05.

## Results

### rhEpo increases red blood cell volume and maximal oxygen uptake in normoxia

The resting hematological changes observed among our rhEpo-treated subjects have been reported elsewhere [6]. Briefly, rhEpo was found to increase red blood cell volume by 9.4% after 5 weeks and by 8.1% after 13 weeks. This was associated with an increase in CaO_2_ of 15.7% after 5 weeks and of 14.5% after 13 weeks of rhEpo treatment.

**Figure 3:**
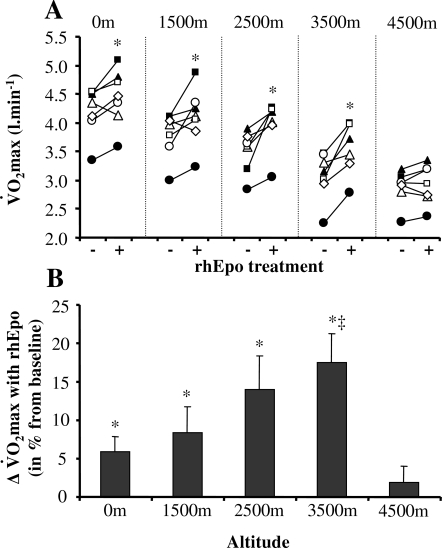
The effect of rhEpo on V̇O_2_max depends on the O_2_ inspiration fraction. This figure refers to the non-invasive study. Panel A shows the individual V̇O_2_max values measured before (−) and after (+) a 5-week treatment with recombinant human erythropoieitin (rhEpo), during normoxia and acute hypoxia equivalent to altitudes ranging between 1500 m and 4500 m. Panel B indicates the changes in V̇O_2_max induced by rhEpo, for each altitude condition. Values are means±SE in seven subjects. *P<0.05 after *vs* before rhEpo. ‡P<0.05 hypoxia (3500 m) *vs* normoxia (0 m).

**Figure 4:**
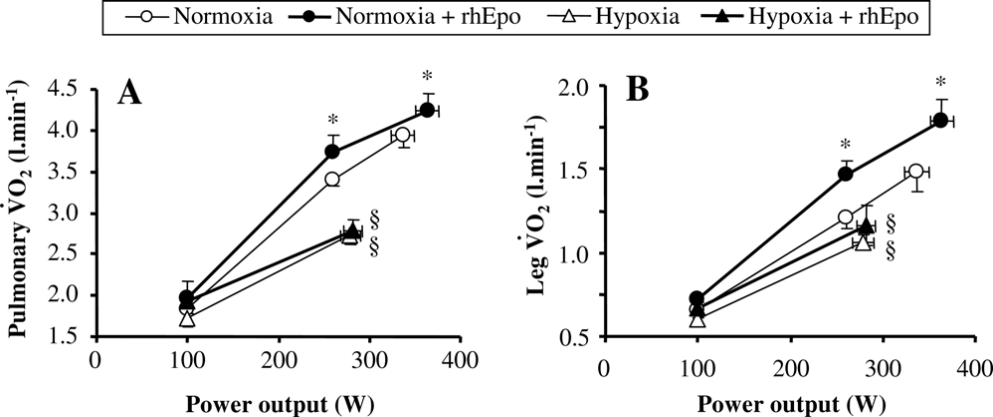
rhEpo increases peak pulmonary and leg V̇O_2_ in normoxia but not in severe acute hypoxia. This figure refers to the invasive study. Pulmonary V̇O_2_ (panel A) and leg V̇O_2_ (panel B) are plotted against power output in the four experimental conditions, i.e. before and after a 14-week treatment with recombinant human erythropoieitin (rhEpo) during normoxia and acute hypoxia equivalent to an altitude of 4500 m. Values are means±SE in seven subjects. *P<0.05 after *vs* before rhEpo. §P<0.05 hypoxia *vs* normoxia.

The ergogenic effect of rhEpo in normoxia is demonstrated from the present data by the increase in maximal oxygen transport 1) at the systemic level with an augmentation of pulmonary V̇O_2_max of 5.8% after 5 weeks (Fig. 3A and 3B) and of 7.2% after 14 weeks of treatment (Fig. 4A), and 2) at the level of exercising muscles, as shown by the concomitant increase in peak leg V̇O_2_ after 14 weeks of rhEpo treatment (Fig. 4B).

### rhEpo increases maximal oxygen uptake during acute moderate hypoxia (1500–3500 m)

Our non-invasive study reveals that after 5 weeks of rhEpo treatment the improvement in pulmonary V̇O_2_max observed in normoxia, of 5.8%, did not decline with increasing levels of inspired hypoxia up to 3500 m (Fig. 3A and 3B). Our results instead suggest that the stimulating effect of rhEpo on maximal oxygen transport was even more pronounced during moderate hypoxia, as the gain in V̇O_2_max reached 14.0% at 2500 m (P = 0.09 *vs* normoxia) and 17.5% at 3500 m (P<0.05 *vs* normoxia) (Fig. 3B). Although the absence of any leg oxygen transport measurement in this part of the study did not allow us to unravel the underlying mechanisms, our results at least indicate that this unexpectedly large gain in V̇O_2_max during moderate hypoxia was related neither to a higher maximal cardiac output nor to a larger rise in CaO_2_ following rhEpo in this condition (Table 1).

**Table 1:** Maximal exercise variables during the non-invasive experiment.

		Maximal exercise				
		Normoxia	Hypoxia			
		0 m	1500 m	2500 m	3500 m	4500 m
Power output, W	**PRE**	362±13	349±14^§^	333±12^§^	309±11^§^	286±12^§^
	**POST**	369±13	360±14	341±16^§^	314±14^§^	287±12^§^
Cardiac output, l.min^−1^	**PRE**	27.0±1.2	26.5±1.2	26.4±0.8	26.2±1.0	25.8±1.1
	**POST**	28.3±1.1	28.0±1.2	28.4±1.3	27.7±1.3	26.8±1.1^§^
Hematocrit^a^, %	**PRE**	46.9±1.4	47.7±1.5	48.2±1.4	47.4±1.4	48.1±1.5
	**POST**	50.2±1.7*	51.9±1.5*	51.4±1.4*	52.3±1.5*	50.8±1.2*
[Hb]^a^, g.dl^−1^	**PRE**	15.3±0.4	15.5±0.5	15.8±0.5	15.6±0.5	15.5±0.5
	**POST**	17.0±0.4*	17.1±0.5*	16.8±0.5*	16.9±0.5*	16.8±0.4*
SaO_2_^a^, %	**PRE**	97.7±0.3	90.2±2.5	85.2±0.8^§^	77.4±2.5^§^	70.6±1.3^§^
	**POST**	96.8±0.7	91.0±1.9	85.4±1.6^§^	79.2±1.1^§^	68.3±1.7^§^
CaO_2_^a^, ml.l^−1^	**PRE**	197.5±6.0	186.7±10.2	180.1±6.8^§^	161.7±10.4^§^	147.1±7.1^§^
	**POST**	220.3±7.0*	206.6±10.5*	190.6±6.3^§^*	179.5±8.0^§^*	153.2±6.4^§^
Lactate^v^, mmol.l^−1^	**PRE**	13.2±0.8	12.6±0.9	12.5±0.5	13.3±0.9	12.4±0.6
	**POST**	12.6±0.4	11.8±1.1	11.6±0.7	12.9±1.3	11.8±0.9
RER	**PRE**	1.14±0.02	1.24±0.02^§^	1.24±0.02^§^	1.24±0.02^§^	1.28±0.01^§^
	**POST**	1.11±0.03	1.18±0.02^§^	1.20±0.02^§^	1.18±0.03^§^	1.33±0.04^§^
Ventilation, l.min^−1^	**PRE**	157±10	156±12	156±10	157±10	147±9
	**POST**	154±8	162±9	160±12	160±10	149±8

Values are means±SE (n = 7). ^a^ reflects arterialized samples from capillary blood; ^v^ reflects arm venous samples. RER, respiratory exchange ratio. The subjects were evaluated before (PRE) and after a 5-week treatment with recombinant human Epo (POST), in normoxia and in acute normobaric hypoxia equivalent to altitudes ranging from 1500 m to 4500 m. ^*^P<0.05 POST *vs* PRE. ^§^P<0.05 hypoxia *vs* normoxia.

### rhEpo does not increase maximal oxygen uptake during acute severe hypoxia (4500 m)

Our results demonstrate that the potential for rhEpo to improve maximal oxygen transport, evidenced both in normoxia and during moderate hypoxia up to 3500 m, evanished during a more severe hypoxic challenge equivalent to 4500 m. This finding appears to be independent of the duration of rhEpo treatment, as our data showed the same insignificant change in pulmonary V̇O_2_max, of 1.9% after 5 weeks (Fig. 3A and 3B) and of 2.0% after 14 weeks of treatment (Fig. 3A). The demonstration that active muscles failed to utilize more oxygen after rhEpo in severe hypoxia of 4500 m comes from the observation of an unchanged peak leg V̇O_2_ in this condition (Fig. 4B). Taken together, our data thus suggest that the minimal level of inspired hypoxia required to abolish the ergogenic properties of rhEpo is above 3500 m and below 4500 m.

### Why does rhEpo fail to improve maximal oxygen uptake during acute severe hypoxia?

The fact that V̇O_2_max did not increase during hypoxia (4500 m) following rhEpo treatment in spite of elevated CaO_2_ levels raises the question of the mechanisms involved. The results here below, obtained from our invasive study, provide some insights into these mechanisms.

#### Pulmonary gas exchange

Peak pulmonary ventilation slightly increased after rhEpo in hypoxia (Table 3), without any concomitant improvement in alveolar PO_2_ (Table 2). Alveolar-to-arterial O_2_ difference reached its highest value during maximal exercise in hypoxia, regardless of rhEpo treatment. By contrast, in normoxia, peak alveolar-to-arterial O_2_ difference tended to decrease (P = 0.06) after rhEpo (Table 2). Finally, SaO_2_ was not altered by rhEpo treatment, either during normoxia or during hypoxia (Table 2).

**Table 2:** Blood oxygenation during the invasive experiment.

		Submaximal exercise			Maximal exercise	
		Normoxia	Hypoxia (4500 m)	Normoxia	Normoxia	Hypoxia (4500 m)
Power output, W	**PRE**	100	100	260	337±13	279±11^§^
	**POST**	100	100	260	364±13	281±10^§^
PAO_2_, mmHg	**PRE**	106.6±0.8	50.3±1.6^§^	107.7±1.3	112.0±0.8	56.9±1.6^§^
	**POST**	105.6±0.9	48.9±1.2^§^	107.5±0.8	111.6±0.8	56.2±0.7^§^
PaO_2_, mmHg	**PRE**	102.4±1.3	37.7±1.3^§^	99.2±1.0	100.1±1.2	40.4±1.0^§^
	**POST**	103.9±1.1	38.1±1.0^§^	99.7±2.1	103.1±2.4	41.0±1.1^§^
A-aDO_2_, mmHg	**PRE**	4.3±0.8	13.3±1.0^§^	8.5±1.4	11.9±1.4	16.5±1.8^§^
	**POST**	1.7±1.6	11.2±1.8^§^	7.8±2.4	8.5±2.5	16.1±1.2^§^
P_fv_O_2_, mmHg	**PRE**	21.0±0.6	13.6±1.0^§^	18.6±1.0	17.4±0.8	10.8±1.5^§^
	**POST**	21.5±0.7	14.0±1.0^§^	20.2±0.9	19.4±0.8	11.8±1.6^§^
SaO_2_, %	**PRE**	98.4±0.1	71.0±2.3^§^	97.5±0.2	96.9±0.4	71.1±1.6^§^
	**POST**	98.1±0.1	70.0±1.6^§^	97.0±0.2	96.6±0.5	70.0±1.4^§^
S_fv_O_2_, %	**PRE**	28.9±1.8	15.3±1.9^§^	17.9±2.4	11.0±3.1	7.5±2.2^§^
	**POST**	27.8±1.7	15.2±2.5^§^	18.9±2.0	11.3±1.8	7.1±1.8^§^
CaO_2_, ml.l^−1^	**PRE**	189.7±5.3	139.7±5.9^§^	189.7±4.2	191.7±4.0	138.1±5.2^§^
	**POST**	209.9±5.3*	151.7±6.5^§^*	209.5±5.7*	210.5±5.2*	153.9±6.1^§^*
C_fv_O_2_, ml.l^−1^	**PRE**	55.6±4.2	30.5±4.1^§^	37.5±5.2	29.0±5.3	17.4±4.6^§^
	**POST**	59.5±4.7	32.2±6.0^§^	42.1±5.0	33.3±4.6	19.4±4.3^§^
[Hb]_a_, g.dl^−1^	**PRE**	14.1±0.4	14.6±0.4	14.3±0.3	14.7±0.4	15.0±0.3
	**POST**	15.6±0.4*	15.9±0.5*	15.9±0.4*	16.3±0.3*	16.2±0.5*
[Hb]_fv_, g.dl^−1^	**PRE**	14.2±0.4	14.5±0.5	14.4±0.4	14.8±0.4	14.8±0.3
	**POST**	15.8±0.4*	15.9±0.6*	15.9±0.4*	15.8±0.5*	16.2±0.5*
Hct_a_, %	**PRE**	43.3±1.3	44.6±1.4	43.9±1.0	44.9±1.1	45.8±0.9
	**POST**	47.8±1.2*	48.5±1.5*	47.5±1.0*	49.8±0.8*	49.5±1.6*
Hct_fv_, %	**PRE**	43.5±1.3	44.7±1.4	43.5±1.0	45.3±1.3	45.4±0.9
	**POST**	48.2±1.2*	49.0±1.8*	48.6±1.4*	48.5±1.5*	49.4±1.5*

Values are means±SE (n = 7). A, alveolar; a, arterial; fv, femoral vein; A-aDO_2_ alveolar-arterial O_2_ difference. The subjects were evaluated before (PRE) and after a 14-week treatment with recombinant human Epo (POST), each time in normoxia and in acute normobaric hypoxia equivalent to 4500 m of altitude. ^*^P<0.05 POST *vs* PRE. ^§^P<0.05 hypoxia *vs* normoxia.

**Table 3:** Acid-base balance, lactate, respiratory exchange ratio and ventilation during the invasive experiment.

		Submaximal exercise			Maximal exercise	
		Normoxia	Hypoxia (4500 m)	Normoxia	Normoxia	Hypoxia (4500 m)
Power output, W	**PRE**	100	100	260	337±13	279±11^§^
	**POST**	100	100	260	364±13	281±10^§^
pH_a_	**PRE**	7.401±.005	7.433±.006^§^	7.351±.007	7.302±.015	7.355±.019^§^
	**POST**	7.394±.004	7.428±.016^§^	7.323±.012	7.295±.011	7.367±.016^§^
pH_fv_	**PRE**	7.314±.004	7.359±.006^§^	7.218±.015	7.136±.026	7.208±.031^§^
	**POST**	7.297±.005	7.347±.017^§^	7.179±.015	7.122±.014	7.212±.022^§^
PaCO_2_, mmHg	**PRE**	37.3±0.9	32.3±1.1^§^	39.8±1.4	38.7±1.2	30.4±1.3^§^
	**POST**	39.2±0.8*	33.3±1.2^§^	41.4±0.9*	39.3±1.0	30.9±0.9^§^
P_fv_CO_2_, mmHg	**PRE**	55.5±1.5	45.5±1.0^§^	66.7±2.7	78.2±4.9	56.1±3.0^§^
	**POST**	62.1±1.2*	48.9±2.1^§^	78.7±2.8*	84.9±3.5	60.1±3.4^§^
Arterial HCO_3_^−^,	**PRE**	23.5±0.3	22.5±0.3	21.3±0.5	16.4±0.7	16.0±1.0
mmol.l^−1^	**POST**	23.9±0.3	22.4±0.5	20.8±0.5	16.6±0.5	16.4±0.5
Femoral vein HCO_3_^−^,	**PRE**	23.4±0.3	22.3±0.3	20.5±0.6	14.6±1.0	15.1±1.0
mmol.l^−1^	**POST**	23.9±0.3	22.7±0.5	20.2±0.4	15.0±0.5	15.4±0.4
Lactate_a_, mmol.l^−1^	**PRE**	1.0±0.1	2.5±0.2	3.7±0.4	10.8±1.4	12.3±1.6
	**POST**	1.2±0.1	3.0±0.4	5.1±0.6	11.4±0.5	11.7±0.9
Lactate_fv_, mmol.l^−1^	**PRE**	1.0±0.1	2.7±0.2	4.4±0.5	12.8±1.9	12.3±1.6
	**POST**	1.2±0.1	3.1±0.4	5.9±0.6	12.7±0.7	13.2±1.1
RER	**PRE**	0.87±0.01	1.01±0.05^§^	0.99±0.02	1.14±0.03	1.26±0.08^§^
	**POST**	0.91±0.01	1.00±0.02^§^	1.04±0.02	1.14±0.02	1.30±0.04^§^
Ventilation, l.min^−1^	**PRE**	44±2	55±3^§^	93±5	150±8	134±8^§^
	**POST**	50±1	63±1^§^*	106±5*	160±6	152±8^§^*
V̇_E_ / V̇O_2_	**PRE**	24±1	34±2^§^	28±2	38±1	49±2^§^
	**POST**	25±1	33±2^§^	29±2	38±1	55±2^§^*
V̇_E_ / V̇CO_2_	**PRE**	28±1	33±1^§^	27±1	33±1	40±2^§^
	**POST**	28±1	33±1^§^	27±1	33±1	41±1^§^

Values are means±SE (n = 7). a, arterial; fv, femoral vein; V̇_E_/V̇O_2_ and V̇_E_/V̇CO_2_, ventilatory equivalents for O_2_ and CO_2_, respectively. RER, respiratory exchange ratio. The subjects were evaluated before (PRE) and after a 14-week treatment with recombinant human Epo (POST), each time in normoxia and in acute normobaric hypoxia equivalent to 4500 m of altitude. ^*^P<0.05 POST *vs* PRE. ^§^P<0.05 hypoxia *vs* normoxia.

#### Systemic oxygen transport

**Figure 5:**
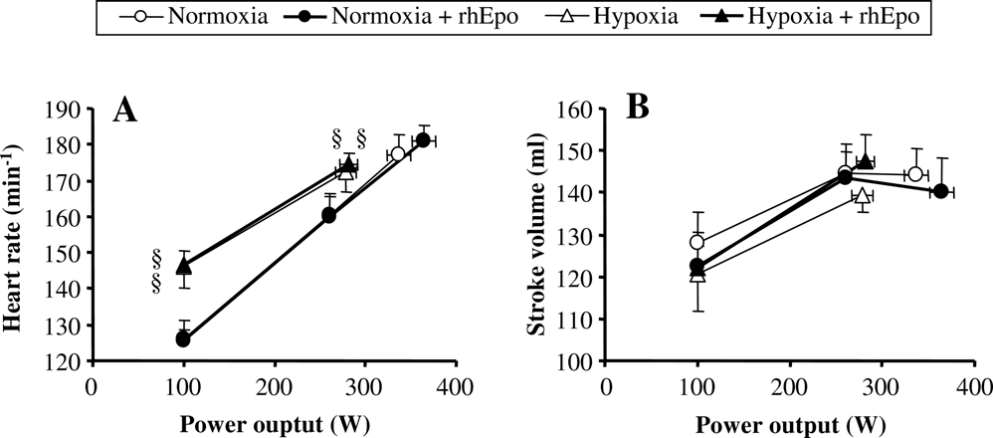
Heart rate and stroke volume after rhEpo in normoxia and in severe acute hypoxia. This figure refers to the invasive study. Heart rate (panel A) and stroke volume (panel B) are plotted against power output in the four experimental conditions, i.e. before and after a 14-week treatment with recombinant human erythropoieitin (rhEpo) during normoxia and acute hypoxia equivalent to an altitude of 4500 m. Values are means±SE in seven subjects.

**Figure 6:**
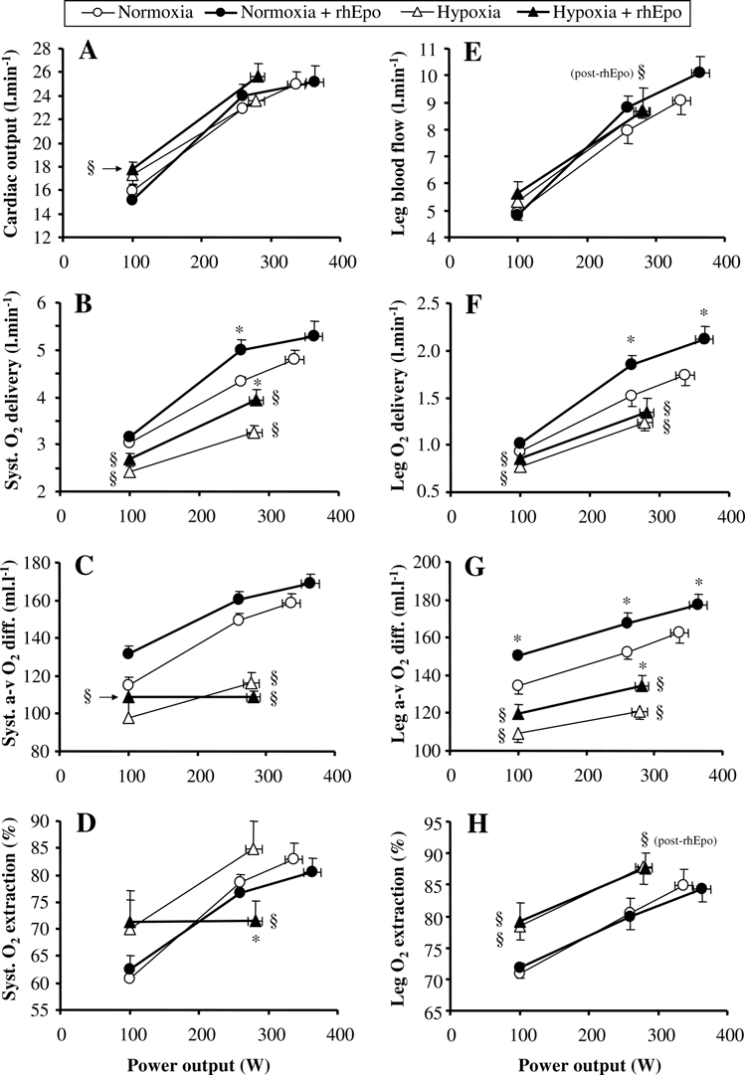
Systemic and leg O_2_ transport variables after rhEpo in normoxia and in severe acute hypoxia. This figure refers to the invasive study. Cardiac output (panel A), systemic O_2_ delivery (panel B), systemic arterio-venous O_2_ difference (Syst. A-v O_2_ diff.) (panel C), systemic O_2_ extraction (panel D), leg blood flow (panel E), leg O_2_ delivery (panel F), leg arterio-venous O_2_ difference (Leg a-v0_2_ diff) (panel G) and leg O_2_ extraction (panel H) are plotted against power output in the four experimental conditions, i.e. before and after a 14-week treatment with recombinant human erythropoieitin (rhEpo) during normoxia and acute hypoxia equivalent to an altitude of 4500 m. Values are means±SE in seven subjects. *P<0.05 after *vs* before rhEpo. §P<0.05 hypoxia *vs* normoxia.

In normoxia, neither maximal heart rate nor maximal stroke volume was affected by rhEpo and this was also true in hypoxia (Fig. 5A and 5B). As a consequence, maximal cardiac output was found similar in all conditions (Fig. 6A). Whatever the oxygenation condition, CaO_2_ was increased after rhEpo (Table 2) resulting in a higher maximal systemic O_2_ delivery both in normoxia (P = 0.06) and in hypoxia (P<0.05) (Fig. 6B). Systemic arterio-venous O_2_ difference, although not wider following rhEpo, increased systematically as power output rose, except during hypoxic exercise (Fig. 6C). Similarly, systemic fractional O_2_ extraction failed to increase along with power output in hypoxia after rhEpo, so that the calculated value during hypoxic exercise was 16% lower than the corresponding value obtained before rhEpo treatment (Fig. 6D).

#### Leg oxygen delivery

Peak leg blood flow was not altered, either by rhEpo or by hypoxia (Fig. 6E). Peak leg O_2_ delivery increased after rhEpo in normoxia only (Fig. 6F). Because CaO_2_ was augmented after rhEpo without any change in C_fv_O_2_ (Table 2), leg arterio-venous O_2_ difference rose after rhEpo independently of oxygenation (Fig. 6G). Finally, leg fractional O_2_ extraction was not altered by rhEpo (Fig. 6H).

#### Distribution of cardiac output

**Figure 7:**
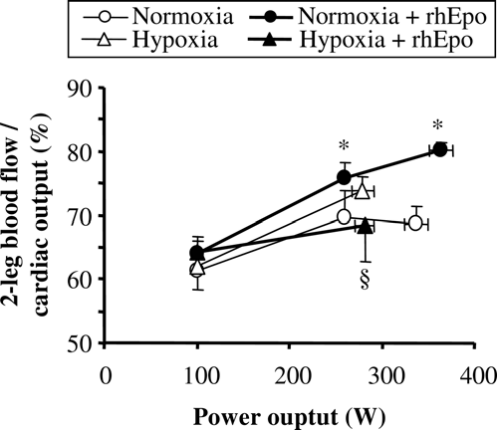
Following rhEpo, blood flow redistribution occurs in normoxia but not in severe acute hypoxia. This figure refers to the invasive study. Two-leg blood flow over cardiac output is plotted against power output in the four experimental conditions, i.e. before and after a 14-week treatment with recombinant human erythropoieitin (rhEpo) during normoxia and acute hypoxia equivalent to an altitude of 4500 m. Values are means±SE in seven subjects. *P<0.05 after *vs* before rhEpo. §P<0.05 hypoxia *vs* normoxia.

Although neither maximal cardiac output nor leg blood flow were significantly altered by rhEpo, Fig. 7 reveals that the distribution of maximal cardiac output was altered, as the fraction of peak cardiac output directed to the exercising legs was 17% higher after rhEpo in normoxia. Conversely, such a change in blood flow distribution did not occur after rhEpo in hypoxia.

#### Blood pressure and vascular conductance

**Figure 8:**
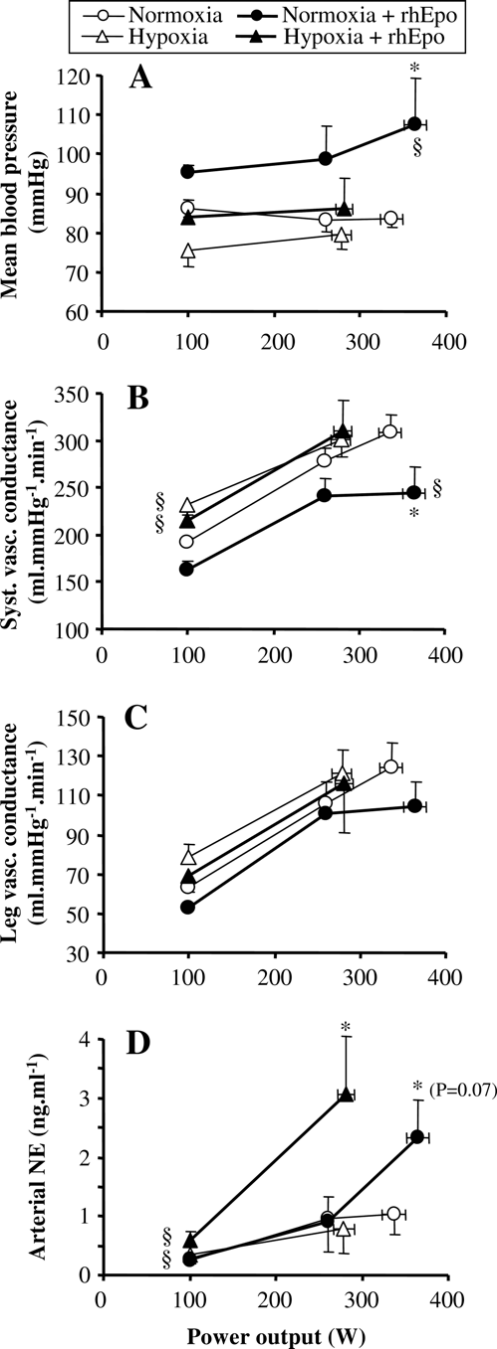
rhEpo increases mean blood pressure and arterial norepinephrine concentration during normoxic exercise. This figure refers to the invasive study. Mean blood pressure (panel A), systemic vascular conductance (panel B), leg vascular conductance (panel C) and arterial plasma norepinephrine concentration (panel D) are plotted against power output in the four experimental conditions, i.e. before and after a 14-week treatment with recombinant human erythropoieitin (rhEpo) during normoxia and acute hypoxia equivalent to an altitude of 4500 m. Values are means±SE in seven subjects. *P<0.05 after *vs* before rhEpo. §P<0.05 hypoxia *vs* normoxia.

Mean arterial blood pressure during maximal exercise was found to be higher following rhEpo in normoxia, but not in hypoxia (Fig. 8A). The same applied for systolic and diastolic blood pressures (results not shown). Systemic vascular conductance during peak exercise decreased after rhEpo in normoxia, while this parameter was not altered by rhEpo in hypoxia (Fig. 8B). Following rhEpo, peak leg vascular conductance was not affected, regardless of oxygenation (Fig. 8C).

#### Acid-base balance, lactate and norepinephrine concentrations in blood

Acid-base balance and blood lactate concentrations are presented in Tables 1 and 3. The levels of arm venous lactate (Table 1), as well as the levels of arterial and femoral venous lactate (Table 3) and potassium (result not shown) measured during peak exercise were similar in all the experimental conditions. Furthermore, rhEpo did not induce any change in leg lactate release (result not shown), regardless of oxygenation. Finally, leg potassium release during peak exercise was found to be similar in all the conditions (result not shown).

Of note is that rhEpo treatment was associated with a large increase in arterial plasma norepinephrine during maximal exercise, reaching 124% in normoxia and 286% in hypoxia compared to the values observed before rhEpo treatment (Fig. 8D).

## Discussion

This study demonstrates that the well-known V̇O_2_max-enhancing effect of rhEpo treatment during exercise in normoxia is also present during mild to moderate hypoxia up to 3500 m, but at 4500 m there is no benefit for V̇O_2_max from rhEpo treatment. This finding implies that there is a threshold altitude between 3500 and 4500 m, from which little or no benefit should be expected from an increased hemoglobin concentration. The main mechanism responsible for this phenomenon resides on the distribution of cardiac output between active muscles and the rest of the body. During exercise in normoxia, the administration of rhEpo is associated with higher CaO_2_ and systemic oxygen delivery combined with a higher fraction of the cardiac output directed to the legs, leading to higher peak leg O_2_ delivery and V̇O_2_. During exercise in hypoxia CaO_2_ and systemic oxygen delivery were also higher after rhEpo treatment, however the fraction of the cardiac output directed to the legs was not increased concurrently, leaving the increased CaO_2_ *per se* insufficient to improve leg maximal oxygen delivery.

Because our study addresses the question of the interactions between hypoxia, CaO_2_ and V̇O_2_max, a prerequisite for data interpretation is that augmented CaO_2_ is the main, if not unique factor by which rhEpo might alter V̇O_2_max. In support of this hypothesis, we separately demonstrated in our rhEpo-treated subjects that if the rise in CaO_2_ associated with rhEpo treatment was acutely reversed by isovolemic hemodilution, the gain in V̇O_2_max conferred by rhEpo was concurrently abolished [25].

### rhEpo improves systemic maximal oxygen uptake during moderate hypoxia

Following rhEpo treatment V̇O_2_max was consistently improved during moderate hypoxia, but not during severe hypoxia. To our knowledge, only one research group has previously evaluated the effect of an acute rise in CaO_2_ and red blood cell volume on V̇O_2_max during moderate acute hypoxia. Their studies showed that after autologous blood re-infusion V̇O_2_max was increased by 9.3% at 2278 m [26] and by 13% at 3566 m [27]. It has also recently been reported that at moderate altitude of 2340 m, the 15% increase in hemoglobin concentration induced by three weeks of acclimatization in athletes resulted in a progressive rise in CaO_2_ that was associated with a 8.9% increase in V̇O_2_max from acute to chronic exposure to 2340 m [28]. Although hemoglobin concentration rose only by 11% after five weeks in our rhEpo-treated subjects, these previous data are nonetheless in line with the present finding showing that increasing CaO_2_ is beneficial for aerobic performance in moderate hypoxia.

A surprising observation was that the improvement in V̇O_2_max was even larger during moderate hypoxia (∼15%) than during normoxia (∼6%) following rhEpo. The observation of a similar V̇O_2_max response at two consecutive levels of simulated altitudes (2500 m and 3500 m) further supports the plausibility of our observation. Our results indicate that in normoxia, the improvement in V̇O_2_max was smaller than the concomitant rise in O_2_ delivery (∼18%), while during moderate hypoxia (F_I_O_2_ = 0.134–0.153), the improvement in V̇O_2_max matched the increase in O_2_ delivery (∼15%). Whatever the mechanism underlying this tighter coupling might be, we suggest that O_2_ utilization responds more “efficiently” to higher O_2_ supply when the O_2_ transport system must cope with moderate arterial hypoxemia.

### In severe hypoxia V̇O_2_max is not increased by rhEpo treatment: potential mechanisms involved

The invasive and non-invasive experiments performed in this study show that rhEpo treatment had no positive effect on V̇O_2_max during severe acute hypoxia. This confirms recent data from our laboratory, showing no increase in V̇O_2_max at 4100 m following prolonged rhEpo treatment [9], as well as earlier evidence demonstrating no augmentation in V̇O_2_max at 4300 m after autologous blood re-infusion [10]. The present finding is also in agreement with high-altitude studies, where V̇O_2_max is classically found barely changed with altitude acclimatization (>4000 m), in spite of a large improvement in CaO_2_ [11], [12], [29]. Although in both situations (severe acute hypoxia after rhEpo *or* chronic hypoxia) V̇O_2_max appears to be independent of the enhancement of CaO_2_, the underlying mechanisms are different. Indeed, prolonged rhEpo treatment stimulates erythropoiesis without increasing hypoxia-inducible factor (HIF-1) levels, while during prolonged hypoxia HIF-1 accumulation triggers Epo gene activation therefore promoting erythropoiesis but also targets many other genes involved in various physiological responses, which may also have a negative effect on V̇O_2_max. In this regard it has been shown that transgenic mice lacking prolyl hydroxylases which otherwise always have HIF-1α downregulate aerobic potential while increasing glycolytic capacity [30].

One major difference between the present data and altitude-acclimatization studies relies on the responses of cardiac output and leg blood flow. With chronic exposure to hypobaric hypoxia (>4000 m), maximal cardiac output is blunted [31] and this is thought to be a major cause of the failure to recover V̇O_2_max after acclimatization [12]. Peak leg blood flow is also decreased with acclimatization [11], [13], not only because cardiac output is lower, but also because blood flow is preferentially directed toward non-exercising tissues [12]. By contrast, following rhEpo maximal cardiac output and leg blood flow were found to be preserved in acute severe hypoxia, in spite of elevated CaO_2_. Such cardiac output response was in line with other data showing no change in maximal cardiac output during acute hypoxia of similar severity (4000–4500 m) without CaO_2_ manipulation [32], [33]. Even so, rhEpo failed to increase V̇O_2_max in severe hypoxia, thus raising the question of the mechanism(s) abolishing the potential advantage conferred by high O_2_ transport capacity.

#### Blood flow redistribution

One possible mechanism, supported by our experimental data, is related to blood flow regulation. In normoxia, rhEpo treatment allowed for a higher fraction of maximal cardiac output to be directed toward exercising muscles, resulting in higher peak leg O_2_ delivery and leg V̇O_2_. Although this blood flow priority given to the active tissue implied a flow limitation into the other vascular beds, it is suggested that oxygenation in non-leg tissues - due to higher CaO_2_- was nevertheless sufficient to allow for vital organs such as heart, respiratory muscles or brain to ensure their homeostasis. Since at maximal exercise there is a competition between tissues for a limited amount of O_2_, a strong activation of the sympathetic system is required to avoid hypotension [34] and maintain an efficient match between O_2_ demand and blood flow distribution [13]. The higher norepinephrine concentrations in plasma during normoxic exercise after rhEpo compared to the corresponding value before rhEpo is compatible with increased sympathetic activation [35] helping to direct a greater fraction of the cardiac output to the legs.

However, the distribution of blood flow during exercise in severe acute hypoxia was not altered by rhEpo, leaving peak leg O_2_ delivery and leg V̇O_2_ unchanged. It is worth noting that at that time, systemic fractional O_2_ extraction was reduced, unlike leg O_2_ delivery, therefore suggesting a limitation in O_2_ extraction into non-leg tissues. Although we could not establish any causal relationship between systemic O_2_ extraction and blood flow redistribution, we speculate that the lack of blood flow redistribution toward exercising muscles following rhEpo in hypoxia could be related to the low O_2_ extraction observed in non-leg tissues. Had blood flow decreased to non-leg tissue areas also in hypoxia, where O_2_ extraction was already diminished in spite of arterial hypoxemia, it is possible that O_2_ delivery/utilization in some organs (i.e. brain, heart or respiratory muscles) and hence local O_2_ homeostasis would have been compromised. Beyond the causes of this lower systemic O_2_ extraction, which remain unclear, our data showed an increase in mixed venous O_2_ content during hypoxic peak exercise, from 22 ml.l^−1^ before rhEpo to 45 ml.l^−1^ after rhEpo, suggesting higher central venous oxygenation levels. Pulmonary gas exchange was not altered by the increase in hemoglobin concentration elicited by rhEpo treatment, and hence, alveolar-to-arterial O_2_ difference and arterial O_2_ saturation were similar to those observed before rhEpo. In agreement with this finding isovolemic hemodilution at altitude did not alter pulmonary gas exchange in altitude-acclimatized subjects [36].

Data on blood pressure and vascular conductance can also shed light on leg *versus* systemic cardiovascular events that occurred during maximal exercise following rhEpo. Indeed, we found that blood pressure during normoxic maximal exercise increased after rhEpo. Since such response was abolished after isovolemic hemodilution [25], increased viscosity due to high hematocrit was a likely candidate of this higher blood pressure, although a direct rhEpo vasoconstrictor effect cannot be ruled out [37]. In association with higher blood pressure, we found that plasma norepinephrine concentration at peak exercise reached higher levels following rhEpo, independently of oxygenation. Although we cannot deduce any causal link between circulating norepinephrine and blood pressure from the present data, one possibility is that the high levels of plasma norepinephrine reflected an overall sympathetically-mediated vasoconstriction [35] which could be the cause of elevated blood pressure. If so, the fact that blood pressure did not increase similarly during hypoxic exercise in spite of elevated norepinephrine could be related to hypoxia itself, which is known to attenuate sympathetic vasoconstriction [38]. Nevertheless, the reason why norepinephrine was higher following rhEpo remains unclear. A direct effect of rhEpo on norepinephrine can be speculated, but has not been verified so far.

In summary, our data indicate that the reason why rhEpo does not boost V̇O_2_max in severe acute hypoxia are: 1) a redistribution of blood flow causing a lower peak leg blood flow following rhEpo (see Fig. 6E and 7), such that O_2_ delivery to the legs in severe hypoxia did not benefit from the increase in CaO_2_ brought up by rhEpo treatment, and 2) a lower systemic O_2_ extraction.

### Insights from the switch between moderate and severe acute hypoxia

One major finding of the present study is that the gain in V̇O_2_max following rhEpo evanished within 1000 m of altitude, i.e. that rhEpo was able blunt V̇O_2_max decrement efficiently during moderate hypoxia (F_I_O_2_ = 0.134) but failed to do so during slightly more severe hypoxia (F_I_O_2_ = 0.115). One previous study is highly relevant to the present one, because it showed a dominance of central fatigue over peripheral muscle fatigue in influencing exercise performance, only if arterial saturation was below 70–75% [39]. Accordingly, we suggest that until the simulated altitude of 3500 m, corresponding to SaO_2_ of ∼78%, task failure in our subjects was likely more dependent on peripheral than central factors, thus enabling increased CaO_2_ to exert its maximal influence on V̇O_2_max. By contrast, during exposure to a higher altitude of 4500 m, SaO_2_ of ∼70% was likely low enough to trigger central nervous system (CNS) hypoxia and central fatigue in our subjects, therefore accounting – at least in part – for our results. Nevertheless, since this previous study [39] did not manipulate CaO_2_ independently of PaO_2_ (as in the present one), we cannot draw a definitive conclusion.

### Study limitations

The timing difference for the non-invasive study (5 weeks) and the invasive study (14 weeks) during the course of rhEpo administration (see Fig. 1) raises the question of the comparability of the two studies, because of a potential rhEpo time effect. Our previously published data with the same subjects indicate that the hematological changes induced by our rhEpo treatment were of the same magnitude at weeks 5 and 13, as reflected by the similar increases in red blood cell volume and CaO_2_ [6]. Furthermore, the similar gain in pulmonary V̇O_2_max seen either after 5 or after 14 weeks of rhEpo treatment, both in normoxia and hypoxia (4500 m), suggests the attainment of a plateau in the V̇O_2_max response within the first month of treatment, as previously shown during 8 weeks of rhEpo administration [3].

Another point to be mentioned is the validity of the non-invasive impedance technique we used in the present study. We acknowledge that the measurement of cardiac output during strenuous exercise with the thoracic cardiac impedance method is a questionable technique [40], however a recent report testing the same impedance device against the direct Fick method found that cardiac impedance was reproducible (95% confidence interval <400 ml.min^−1^) and provided a clinically acceptable evaluation of cardiac output in healthy subjects during an incremental exercise [18]. Our own comparison of the impedance method against the cardio-green dye dilution technique showed values for bias and 95% confidence interval (see *Methods* and Fig. 2) that were very similar to those previously published [18].

### Conclusion

What was already known in the field is that prolonged rhEpo treatment boosts V̇O_2_max in normoxic conditions but not during severe hypoxia. What the present study adds to the established knowledge is that rhEpo efficiently blunted the decrement of V̇O_2_max up to the altitude of 3500 m, supporting the idea that CaO_2_ is a limiting factor of V̇O_2_max during moderate hypoxia. Furthermore, our study demonstrates that rhEpo-stimulating effect on V̇O_2_max was mediated not only by increased CaO_2_ but also by preferential blood flow redistribution toward exercising muscle tissue. We do not exclude the possibility that this advantageous blood flow redistribution also occurred at moderate altitude, therefore explaining why V̇O_2_max was substantially improved in this condition. That such cardiovascular changes did not take place during severe hypoxia would aim to protect some vital organs from deleterious hypoxemia.
